# Suitability of measures of self-reported medication adherence for routine clinical use: A systematic review

**DOI:** 10.1186/1471-2288-11-149

**Published:** 2011-11-03

**Authors:** Sara Garfield, Sarah Clifford, Lina Eliasson, Nick Barber, Alan Willson

**Affiliations:** 1 The Centre for Medication Safety and Service Quality, The School of Pharmacy, Mezzanine Floor, BMA House, Tavistock Square, UK; 2 Centre for Haematology, Imperial College London, Hammersmith Hospital, Du Cane Road, London W12 0NN, UK; 3 National Leadership and Innovation Agency for Healthcare, Bridgend Road, Llanharan, Glamorgan, UK

## Abstract

**Background:**

There is a recognised need to build primary care medication adherence services which are tailored to patients' needs. Continuous quality improvement of such services requires a regular working method of measuring adherence in order to monitor effectiveness. Self report has been considered the method of choice for clinical use; it is cheap, relatively unobtrusive and able to distinguish between intentional and unintentional non-adherence, which have different underlying causes and therefore require different interventions. A self report adherence measure used in routine clinical practice would ideally be brief, acceptable to patients, valid, reliable, have the ability to distinguish between different types of non-adherence and be able to be completed by or in conjunction with carers where necessary.

**Methods:**

We systematically reviewed the literature in order to identify self report adherence measures currently available which are suitable for primary care and evaluate the extent to which they met the criteria described above. We searched the databases Medline, Embase, International Pharmaceutical Abstracts, Pharmline, CINAHL, PsycINFO and HaPI to identify studies reporting the development, validation or reliability of generic adherence measures. One reviewer screened all abstracts and assessed all relevant full text articles obtained and a second reviewer screened/assessed 10% to check reliability.

**Results:**

Fifty eight measures were identified. While validation data were presented in support of the vast majority of self reported measures (54/58), data for a relatively small number of measures was presented for reliability (16/58) and time to complete (3/58). Few were designed to have the ability to be completed by or in conjunction with carers and few were able to distinguish between different types of non-adherence, which limited their ability be used effectively in the continuous improvement of targeted adherence enhancing interventions. The data available suggested that patients find it easier to estimate general adherence than to report a specific number of doses missed. Visual analogue scales can be easier for patients than other types of scale but are not suitable for telephone administration.

**Conclusions:**

There is a need for a measure which can be used in the routine continual quality monitoring of adherence services.

## Background

There is a need to build informed, comprehensive, primary care-based medication adherence services which are tailored to patients' needs [1]. Non-adherence to medication is estimated to affect approximately 30-50% of patients with chronic conditions [2]. The consequences include a missed opportunity for treatment effect, poor health outcomes and increased healthcare costs. For example, it has been estimated that non-adherence is responsible for 48% of asthma deaths, an 80% increased risk of death in diabetes and a 3.8-fold increased risk of death in the year following a heart attack [3]. In the United Kingdom National Health Service (NHS), medicines are the biggest expenditure after staff and 71% of the medication budget is spent in primary care. It is estimated that the current cost of unused or unwanted medicines exceeds £300 million annually [4]. A recent paper that mapped the quality of medicines use in primary care estimated that only between four and 21% of patients achieve effective, error free care, and that an improvement in adherence would be the most significant area to target to improve medicine use [5].

If one is to improve some aspect of a service or behaviour it is generally accepted that there needs to be some measure associated with the behaviour, which can be used to assess the success of interventions. All the various managerial continuous improvement processes depend on having key measures which can be taken repeatedly as an indicator of the success of improvement strategies. Improving adherence should be no different. In order to build effective targeted adherence services, a simple, valid and reliable method for detecting the prevalence and type of non-adherence to medicines is needed [6]. It is important to be able to monitor the extent of adherence in individual patients and populations as part of routine clinical practice. There is a requirement for a method with which non-adherence can be assessed in individuals, appropriate interventions instigated, and the effectiveness of the intervention evaluated. In order to continually improve quality of services provided to a population, there is a need to have a regular working method to monitor the effectiveness of the service and for pharmacists and Primary Care Trusts (local state funded primary care management organisations) to evaluate the effectiveness of changes to the service. In adherence-related services, improvements in adherence of the population using the service is an important marker of quality. In order to monitor adherence, understand the reasons for non-adherence and improve the effectiveness of adherence services there is a need for frequent, regular measurements which are not very time consuming to make.

As it is not possible to measure actual adherence without continually observing patients, the measures that are used may be considered as proxy measures of adherence rather than absolute measures of adherence. Methods that have been used include using a container that has an electronic chip in the lid that records the time of each opening (Medication Event Monitoring System- MEMS), pill counts, pharmacological and biochemical markers, medical and dispensing records and self report. Each of these measures assesses a different stage in the prescribing and use of medicines, and a different time period. Thus medical records measure the amount of medication prescribed, dispensing records the amount of medication dispensed, MEMS measures the opening of the container, pill counts measure the amount of medication removed from the container, pharmacological markers give an indication of when and how much medication the patient has ingested and self report measures the patients' recall of what they have taken. Medication prescribed, dispensed, the opening or emptying of containers or report that medication has been taken do not necessarily reflect what the patient has taken and therefore may be considered as measures of variables indicative of adherence rather than absolute measures of medication use. However, they are described as adherence measures in the literature, and are described as such in this review for consistency.

Therefore rather than there being a 'gold standard', the choice of measure will depend on the specific situation. Many of the measures used in research have limited feasibility for use in clinical practice. For example, accurate pharmacological measures are only available for a limited number of medications and are intrusive and costly. They may therefore be useful when measuring adherence of a specific medication for which there is a pharmacological indicator to enable change during the intervention to be assessed and when there is a limit to the number of times adherence needs to be measured. MEMS is also very expensive relative to the costs of many medicines used in primary care and is not suitable for all formulations and medications. It may be more useful for a single solid dosage medication being delivered and monitored as part of a clinical trial than multiple drug use in routine primary care. Medical records, dispensing records and pill counts may assist with building a picture of patients' adherence over time but will not be able to assess adherence at a more specific point in time and may not be as useful for continuous routine monitoring.

Self report may be considered the most appropriate method to use for monitoring adherence as part of continuous quality improvement in clinical practice. Self report has its disadvantages as patients are known to overestimate their level of adherence [6]. For this reason in research studies self report is triangulated with other methods of measuring adherence [6]. However, NICE guidelines have identified that whilst other types of measures are useful for clinical trials of new drugs, self report is an appropriate tool for clinical practice [7]. Triangulating between methods would not be practical for regular clinical use. In clinical practice we need to measure adherence in a cheap and relatively unobtrusive way which can be used routinely. We also need to distinguish between intentional and unintentional non-adherence, which have different underlying causes and therefore require different interventions. Self report is the only measure which is able to meet all these criteria. Recent reviews have shown that self report has moderate correlation with electronic monitoring [8,9], although self reported adherence levels are higher than adherence levels derived from MEMS [9].

A self report measure, which can be used for the continuous quality improvement of adherence related services, needs to have optimal pragmatic, psychometric and theoretical properties.

### Pragmatic properties

The measure must be low cost, brief and non intrusive so that it can be used to take repeated measures of self reported adherence over time. It would be preferable for the measure to be able to be administered in different ways, for example, by telephone, face to face and by post. It would also be advantageous if the measure was suitable for both patient self administration and for administration by an interviewer. Questionnaires have been found to have better correlation with other measures than interviews [10] but some patients may require assistance, so flexibility in method of administration is important. In primary care patients are often treated for multiple conditions. Therefore a measure for a primary care adherence service needs to be generic rather than disease specific and should be suitable for patients taking a single medication or multiple medications for different conditions. It is important to include information from carers where appropriate as it is acknowledged they can have significant roles in medication management [11].

### Psychometric properties

Validity (a check that the instrument is measuring what it is supposed to measure) should be established. In adherence research, this is often done by checking the measure against other measures of adherence such as MEMS, Pill counts and clinical markers). Reliability (the consistency of the measurement score) and acceptability to respondent are required. The measure must also be sensitive enough to measure change.

### Theoretical properties

The measure also needs to have a theoretical basis that reflects the need for patient tailored adherence services. The accident causation framework can be used to establish the underlying contextual and individual factors contributing to non-adherence [12]. Thus adherence can be seen as a 'symptom' rather than a 'diagnosis'. In order to target adherence services to support patients' needs, it is necessary to establish the causes of non-adherence. In particular we need to be able to distinguish between intentional and unintentional non-adherence, as they have different underlying causes and therefore require different interventions. In the past, explanatory psychological models (such as the social cognition theories [13] and the stages of change model [14]) have been used to identify beliefs and attitudes that are predictive of non-adherence. However, models focussing on beliefs can only explain intentional behaviour, whilst unintentional causes of non-adherence are left unexplained. Therefore, such models are not suitable for the development of self report measures that are able to diagnose the extent and full range of reasons for non-adherence, in individual patients and populations. The accident causation framework is the only model that we have found that has incorporated unintentional non-adherence.

In this paper, we aim to systematically review the literature in order to identify the self report adherence measures currently available which are suitable for generic use and evaluate the extent to which they meet the theoretical, pragmatic and psychometric criteria described above.

## Methods

### Search strategy

We carried out a systematic review of studies addressing the development, validation and reliability of self reported adherence measures, searching the following databases: Medline (1948-July 2010), EMBASE (1980-July 2010), International Pharmaceutical Abstracts (1970-July 2010), Pharmline (1978-July 2010), CINAHL (1982-July 2010), PsycINFO (1806-July 2010) and HaPI (1985-July 2010). We used the keywords(complian * OR adheren*) AND (measure* OR scale* OR questionnaire*) AND (self AND report* OR self-report*) AND (valid* OR develop*). We also searched the reference lists of relevant papers in order to identify any additional studies and contacted known experts.

### Inclusion and exclusion criteria

#### Inclusion Criteria

Studies that report development, reliability or validation of a retrospective self report adherence measure against a non-questionnaire measure (such as MEMS, pill counts and clinical markers).

#### Exclusion Criteria

• Studies not published in English.

• Studies reporting development or validation of a questionnaire written in a language other than English.

• Studies reporting development or validation of a questionnaire where the full wording of the questionnaire is not available (authors were emailed once to request this if it was not published).

• Studies reporting development or validation of a questionnaire including adherence questions only relevant to specific illnesses or medications (as primary care patients have a wide range of conditions).

• Studies reporting a questionnaire focussing on beliefs about medicines rather than adherence.

• Studies reporting questionnaires including items not related to medication adherence (unless medication adherence items form a separate subscale).

• Studies reporting questionnaires not suitable for multiple medications (as patients in primary care often take more than one medication).

• Studies reporting use of a questionnaire with no description of development or validation or reliability testing.

• Studies only reporting validation against another questionnaire.

• Non peer- reviewed studies.

• Studies which solely include prospective rather than retrospective questionnaires.

• Diary methods (as these are too intrusive in patients' day to day life for routine use).

• Studies where the adherence measure used was not reproducible.

• Studies where the results were not clearly reported.

• Studies where no statistical comparison was reported.

### Screening and data extraction

One reviewer screened all titles and abstracts in order to determine whether the full research paper should be retrieved or whether it was evident it did not meet the inclusion criteria at this stage. A second reviewer independently screened a random 10% sample in order to check the reliability of the screening (the agreement level was 91%). The first reviewer reviewed all retrieved articles to determine whether the article met the inclusion criteria. The second reviewer independently reviewed a random 10% sample of full text papers to check reliability (the agreement level was 93%). In addition, the first and second reviewers discussed all articles which the first reviewer viewed as being borderline between inclusion and exclusion. The first reviewer then extracted data from the included articles. This included number of items, scale type, time period over which adherence is measured, measurement of reasons for non-adherence, description of development of the scale and the sample size, population and results of validity and reliability studies.

## Results and Discussion

### Studies identified

**Figure 1:**
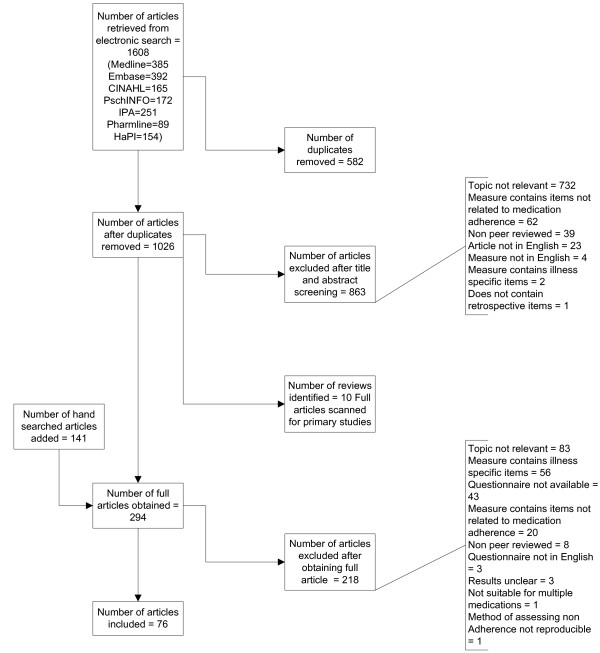
**Flow chart of papers identified, screened and evaluated**.

One thousand and twenty six abstracts were screened and 294 full text articles were obtained. Seventy six papers met the inclusion criteria [15-91](see Figure 1). These included 58 measures. The included scales and properties are shown in additional files 1, 2.

A large amount of recent work on adherence has been carried out in the area of human immunodeficiency virus (HIV) and a variety of measures have been used, many of which have been developed for specific studies. A large number of these did not meet the inclusion criteria as either the measure used was unavailable or it contained items which were not generically relevant, often related to very accurate timing which is of particular importance to HIV medication but not to medication for most other conditions. The original Adult AIDS Clinical Trials Group (AACTG) instrument [92] (designed to measure adherence to HIV medication) contains an item related to accurate timing; hence these papers had to be excluded. However, some studies used the AACTG without the accurate timing item; therefore these studies were included.

### Pragmatic properties of measures

Limited data were available on the acceptability of the included measures to respondents. The length of measures ranged from 1 to 21 items. Where information regarding completion time is presented (3/58), scales were all reported to take under 5 minutes to complete.

A variety of formats were used to administer questionnaires including face to face interview, telephone interview, self administration and computer programmes. Few studies compared different formats. However different methods of administering an adherence measure were compared in two studies [82,89]. Nau [89] found no difference between administering the VAS (visual analogue scale) by mail or a non-visual version by telephone, (the latter relied on asking for percentage without the use of the visual scale). Kalichman [82] compared computer administration of the VAS to telephone administration of the same question without the visual scale. The adherence assessments obtained by telephone correlated with those obtained over the computer (r = 0.7, p < 0.01). However, adherence reported over the telephone was significantly higher (p < 0.01) than that reported by computer assessment. As self report is known to overestimate adherence, these data may suggest that the adherence question used with the VAS is less accurate when used without the visual scale.

The majority of the measures were designed for use with adult patients. However, one measure was used with adolescents [24] and another was designed for use with parents to establish adherence rates with their children's medication [90]. No other measures were reported as having a version which could be completed by carers of patients.

Many of the measures asked about adherence over a specified period of time such as a week or a month (43/58) and would therefore be more suitable for measuring adherence over a specified time period than those that did not specify a time period. Measures that did not specify a time period (15/58) would not be able to demonstrate change when repeated over time.

### Theoretical properties of measures

There was variation in the detail given about the development of scales. Some were described as being developed in conjunction with a literature review and/or with input from patients and/or healthcare professionals. Few were described as being based on an explanatory model. However, one measure was based on the Transtheoretical Stages of Change Theory [24,88] and identified the stage that a patient was at with regard to their medication taking behaviour, ranging from precontemplation (not considering taking medication) to maintenance (taking medication as prescribed). It therefore focused on intentional more than unintentional behaviour. Half the measures incorporated some reasons for not taking medication [15-24,28,29,32-34,36-40,43-45,48,51-53,60-70,74,77,80-82,85-87] but in the vast majority of cases these reasons were not developed in conjunction with interviews with patients and were not necessarily comprehensive. Intentional and unintentional non-adherence were only distinguished from each other in one measure [35].

### Psychometric properties of measures

Data regarding internal and test-retest reliability was presented for a relatively small number of measures [28,29,32,36,39,45,53-55,60-65,74,77,80,86,91] (16/58). Where the data are presented the reliability is generally acceptable (Cronbach alpha 0.6-0.9, ICC and Kappa > 0.7). The exception is that the Morisky scale is reported as having low internal reliability in half the papers that assessed it [36,61,63,65], although the other half reported acceptable reliability [60,62,64,91].

Validation data were presented in support of the vast majority of measures (54/58), although this varied in quality in terms of sampling method, sample size and the level of statistical information reported. Self report measures have been correlated with other measures of adherence: MEMS, pill counts, pharmacy refill data and clinical measures such as viral load and blood pressure. For the majority of studies the sampling strategy used was either not reported or was stated to be a convenience sample. Sample size varied greatly between studies, ranging from 22 to 1985. Eleven studies had a very small sample size (< 50) and were hence unlikely to identify real differences that may have existed between measures; possibly leading to the incorrect conclusion that the test measure was valid.

It is difficult to compare studies due to differences in sample size, sampling strategy, population and the measures correlated against. However, some interesting information can be obtained where two measures have been compared in the same study. Findings of such studies have suggested that patients find it easier to estimate general adherence than to report a specific number of doses missed. Two studies showed that a single question asking about a general adherence pattern over a month had greater validity than asking detailed questions about the amount of medication missed over the last few days when correlated with MEMS [50] pill counts [82] and viral load in HIV patients [82]. However, these studies do not address the issue of whether more detailed questions would gather more information about building a pattern of patients' medication taking behaviour and thereby aid the choice of suitable interventions. In contrast to the above findings [50,82], in one study the relationship between self reported adherence and viral load reached significance when patients were asked about the number of doses missed within the last week but not when they were asked about the last time they missed a dose over a longer period [73]. These findings suggest that it is question type rather than time period which improves accuracy and ease of use. Schneider et al [77] found that patients reported in cognitive tests that it was easier to respond to questions with Likert type responses than to questions that asked about percentages of doses skipped. Lu [50] found that asking patients to rate their ability to take their medication was correlated better with MEMS than asking them how frequently they took them or what percentage of the time they took them. Whilst social desirability may be one factor leading to overestimation of adherence, making questions less judgemental was not found to increase reporting of non-adherence, suggesting that other factors such as memory recall, are important [93].

There was little information in the literature regarding whether dichotomous, Likert or visual analogue scales are more accurate. In one study [36] a Likert version of the Morisky measure was created and both this version and the standard dichotomous version were validated against pill count. No comparative statistical tests between the dichotomous and Likert measures were carried out but the descriptive results indicate they performed similarly against the pill count. In another study a visual analogue scale had higher correlation with psychotropic dose concentration than a likert scale [53]. A consideration is that the level at which non-adherence becomes clinically significant varies between medications. For example a 95% adherence rate is needed for optimal therapeutic benefit of protease inhibitors to control HIV [94], a 90% adherence rate is required for imatinib in the treatment of chronic myeloid leukemia [95] and for many medications the adherence rate needed is unknown. Dichotomous categorizations that rely on arbitrary cut off points such as 80% adherence, maybe less helpful, compared to a scale, when used by health care professionals for continuous monitoring of patients over time in order to increase adherence to a level that allows optimal therapeutic benefit.

### Limitations

The search strategy was designed to identify all adherence/compliance measures described as such by the authors of instruments and papers. If authors incorrectly described an adherence instrument as another type of measure, such as a concordance or persistence measure, this would not have been identified in the review. In addition, studies published after the search was completed would not have been identified.

## Conclusion

Self report has been recommended as the measure most suitable for clinical practice [7]. A large number of self report measures have been used to measure adherence and several measures of adherence have some of the properties that are required to be used in routine clinical practice, particularly validation against non questionnaire measures. However, there is limited information about which types of scales are most acceptable and non intrusive to patients, are the most reliable and obtain the most accurate information. The scales available have limited ability to distinguish between intentional and unintentional non-adherence and few scales allow for the role of carers in medication management. Currently, the most appropriate instrument will need to be selected based on the user's prioritisation of the properties required e.g. responsiveness to change (potentially possible with the 43 measures specifying a time period), established reliability [28,29,32,36,39,45,53-55,60-65,74,77,80,86,91], provision of information on causes [15-24,28,29,32-34,36-40,43-45,48,51-53,60-70,74,77,80-82,85-87] or suitability for completion by parent [90]. There is a need for the development of a measure which can be used in the routine continual quality monitoring of adherence services and which meets all the above criteria.

## Competing interests

The authors declare that they have no competing interests.

## Authors' contributions

SG conducted the literature review and wrote the paper. SC was involved in developing the inclusion and exclusion criteria, acted as the second reviewer and was involved in editing the paper. LE was involved in developing the search strategy and involved in editing the paper. NB was involved in editing the paper. AW secured funding and was involved in the conceptualization of the paper. All authors read and approved the final manuscript.

## Pre-publication history

The pre-publication history for this paper can be accessed here:

http://www.biomedcentral.com/1471-2288/11/149/prepub

## Supplementary Material

Additional file 1 **Properties of adherence scales**. A table of all measures included in the review and their properties including number of items, scale type, time period over which adherence is measured, measurement of reasons for non adherence and acceptability. Click here for file

Additional file 2 **Validity and reliability of self reported adherence scales**. A table giving details of validity and reliability studies on included measures. Click here for file
