# Supplementary material for: Suitability of measures of self-reported medication adherence for routine clinical use: A systematic review
Source: BMC Med Res Methodol. 2011 Nov 3;11:149. doi: 10.1186/1471-2288-11-149 (PMC3219622; doi:10.1186/1471-2288-11-149)
Supplement: Additional file 1 — Properties of adherence scales. A table of all measures included in the review and their properties including number of items, scale type, time period over which adherence is measured, measurement of reasons for non adherence and acceptability. [file 1471-2288-11-149-S1.RTF]

Scale	Number of items 	Scale type	Time period over which adherence is measured	Measurement of reasons for non-adherence 	Description of development of scale	Acceptablity	
Adult AIDS Clinical Trials Group (AACTG) 4 day version [15-18]	4	Continuous	Previous 4 days	Yes, closed set of reasons, not split into intentional and unintentional non-adherence	No description of development	No data presented	
AACTG 3 day version [19-21]	3	Continuous	Previous 3 days	Yes, closed set of reasons, not split into intentional and unintentional non-adherence	No description of development	No data presented	
AACTG last 2 day + Saturday version [22]	3	Continuous	Previous 2 days and previous Saturday	Yes, closed set of reasons, not split into intentional and unintentional non-adherence	No description of development	No data presented	
AACTG 7 day version [23]	1	Continuous	Previous 7 days	Yes, closed set of reasons, not split into intentional and unintentional non-adherence


	No description of development	No data presented	
AACTG  one month version [24]	3	Continuous	Previous month	Yes, closed set of reasons, not split into intentional and unintentional non-adherence	No description of development	No data presented	
Adherence Self Report Questionnaire (ASRQ)  [25-27]	1	Likert	None Specified	No	Based on earlier instrument	80% stated clear wording
83% stated questions not embarrassing
Majority needed less than 2 minutes to complete	
Adherence to Refills and Medication Scale (ARMS) [28]	12	Likert	None specified	Yes, closed set of reasons, not split into intentional and unintentional non-adherence	Based on earlier measures, cognitive interviews with patients and evaluation of the wording by patients	Lexile literacy score 920 (eighth grade higher score = greater difficulty)	
Ask 12 behaviour subscale [29]	5	Dichotomous	Previous month	Yes, closed set of reasons, not split into intentional and unintentional non-adherence	Items generated from comprehensive literature review, expert panel input and patient focus groups
	No data presented	
Barroso [30]	1	Likert	Previous 7 days	No	No description of development	No data presented	
Bell [31]	1 (4 parts)	Dichotomous	Previous day, week, month and ever 	No	No description of development	No data presented	
Brooks Medication Adherence 
Scale [32]	4	Dichotomous	Previous 3 months	Yes, closed set of reasons, not split into intentional and unintentional non-adherence	Modified previous measure to provide more information and a specified time period	No data presented	
Choo [33,34]	5	3 continuous items, 1 dichotomous item and 1 likert scale item	3 items previous week
2 items none specified	Yes. 2 items to measure unintentional non-adherence and 3 items to measure intentional non-adherence	Three of the items derived from previous measure.  Two items added to include adherence not related to a specific time period	No data presented	
Cohn [35]	8	Dichotomous	Previous  24 hours, 48 hours and 2 weeks	No	No description of development	No data presented	
Composite self report measure [36]	5	Likert	None Specified	Yes, patients classified as intentionally or unintentionally non adherent
	Based on earlier measure	No data presented	
CPCRA antiretroviral medication self- report 3 day adherence [37]	3	Likert and dichotomous items	Previous 3 days	Yes, not split into intentional and unintentional non-adherence	No description of development	No data presented	
CPRCA antiretroviral self-report 7 day adherence [37-38]	3	Likert and dichotomous items	Previous 7 days	Yes, not split into intentional and unintentional non-adherence	No description of development	No data presented	
Erickson [39]	4	Likert	Previous 3 months	Yes, closed set of reasons, not split into intentional and unintentional non-adherence	Based on earlier  measure	No data presented	
Fodor [40]	3	Likert	None specified	Yes, closed set of reasons, not split into intentional and unintentional non adherence	Designed to be nonthreatening and avoid negative connotations	No data presented	
Gehi [41]	1	Likert 	Previous month	No	No description of development	No data presented	
Grymonpre [42]	2	Continuous	None specified	No	No description of development	No data presented


	
Hill Briggs [43]	5	Dichotomous	None specified	Yes, closed set of reasons, not split into intentional and unintentional non-adherence	Based on earlier  measure and adapted according to previous research  	No data presented	
Immunosupressant Therapy Adherence Scale (ITAS) [44-45]	5	Likert	Previous 3 months	Some reasons, not split into intentional and unintentional non-adherence	Based on earlier measure, pretested in 25 patients for understanding and modified as appropriate	No data presented	
Inui [46]	 1	Dichotomous	Previous 2 months	No	Based on earlier measure	No data presented	
Kerr [47]	1	Dichotomous	Previous 2 months	No	Based on earlier measure	No data presented	
Leopold [48]	21	Likert	Previous month	Yes, closed set of reasons, not split into intentional and unintentional non-adherence	No description of development	No data presented	
Liu [49]	1	Continuous	Previous 7 days	No	No description of development	No data presented	
Lu 1month frequency scale [50]	1	Likert 	Previous month	No	No description of development	 No data presented	
Lu 1 month Percent scale [50]	1	Likert 	Previous month	No	No description of development	 No data presented	
Lu 1 month rating response scale [50]	1	Likert 	Previous month	No	No description of development	 No data presented	
Multicentre aids cohort study (MACS) adherence form [51]  	5	Continuous and dichotomous items	Previous 4 days	Yes, closed set of reasons, not split into intentional and unintentional non-adherence	Piloted during development	No data presented	
MARS [52-53]	5	Likert	None specified	Yes, closed set of reasons, not split into intentional and unintentional non adherence	No description of development	No data presented	
Medication Adherence Evaluation Scale
(MASS)[54]	6	Continuous	Previous week, month and 12 months	No	Developed to be used in routine clinical practice. Desired properties = short, easy administration and interpretation, measures major and minor non-adherence and  past and present non-adherence.	No data presented	
MATI [18]	1	Continuous	None specified	No	No description of development	No data presented	
Medication adherence self report inventory (MASRI) part A –[55-66]	6	Likert and continuous (visual analogue) items	Previous month	No	Based on literature	No data presented	
Medical Outcomes Study Adherence question [57]	1	Likert	Previous 4 weeks	No	No description of development	No data presented	
Melbourne [58]	1	Likert	None Specified	No	Based on earlier instrument	No data presented	
Mooney [59]	1	Dichotomous	Since last clinic visit	No	No description of development	No data presented	
Morisky 4 [60-68]	4	Dichotomous	None specified	Yes, closed set of reasons, not split into intentional and unintentional non-adherence by Morisky but items 1 and 2 regarded as  intentional and 3 and 4 as non intentional by later user  


	Based on earlier measure and designed to reflect different ways of omitting medication.  Reverse wording of questions to help obtain disclosures of non-adherence.	Lexile literacy score 650L (fourth grade higher score = greater difficulty)	
Morisky 4 likert [36]	4	Likert	None specified	Yes, closed set of reasons, not split into intentional and unintentional non-adherence by Morisky but items 1 and 2 regarded as  intentional and 3 and 4 as non intentional by later user  	Based on earlier measure and designed to reflect different ways of omitting medication.  Reverse wording of questions to help obtain disclosures of non-adherence.	No data presented	
Morisky 8 [69-70]	8	Dichotomous and one likert scale item	One item- previous day, one item previous 2 weeks
Remainder none specified	Yes, closed set of reasons, not split into intentional and unintentional non adherence	Based on earlier  measure and supplemented with additional items addressing the circumstances surrounding adherence behaviour	No data presented	
Murphy 1[71]	2	Likert	Previous month	No	No description of development	No data presented	
Murphy 2 [72]	2	Likert	Previous month	No	No description of development	No data presented	
Prado [67]	1	Dichotomous	Previous month	No	No description of development	No data presented

	
Pratt [73]	1	Continuous (visual analogue)	None Specified	No	No description of development	No data presented	
Reported  adherence to medication (RAM) [74]	4	Likert	Previous 4 days	Yes, closed set of reasons, not split into intentional and unintentional non adherence	No description of development	No data presented	
Regimen screen of Brief Medication Questionnaire (BMQ) [75]	5	Continuous	Previous week	No	Aimed to develop an instrument that was brief and easy to use, detect regular and sporadic non-adherence and be self administered by patients with multiple drugs 	No data presented	
Remington [76]	1	Likert	None specified	No	No description of development	No data presented	
Schneider [77]	4	Likert	Previous 4 weeks	Side effects only	Cognitive testing of several approaches and consultation with national adherence experts
	No data presented	
Schuman[78]	1	Likert	Previous 7 days	No	No description of development	No data presented	
Self reported adherence (SERAD)[79]	15	Continuous	Previous week, month and 3 months	No	No description of development	Took an average 3.7 mins to complete	
Simplified [80-81] Medication Adherence Questionnaire (SMAQ)	6	Dichotomous and continuous items	Previous week and previous 3 months and ever	Yes, closed set of reasons, not split into intentional and unintentional non-adherence	Based on previous measure and adapted to be more adherence specific.  Developed by a multidisciplinary team	No data presented	
7SR Recall [82]	11	Continuous	Previous 7 days	Yes, closed set of reasons, not split into intentional and unintentional non-adherence	Reasons for non-adherence adapted from previous research.  No other description given.	No data presented	
Stages of change (SOC) [83-84]	2	Likert	Over a year	No	Based on stages of change model	No data presented	
Stewart [85]	11	Continuous	Previous 7 days	Yes, closed set of reasons, not split into intentional and unintentional non-adherence	Reasons for non-adherence adapted from previous research.  No other description given.	No data presented	
Tool for Adherence Behaviour screening (TABS) [86]	8	Likert	None specified	Yes, closed set of reasons, not split into intentional and unintentional non-adherence	Based on in depth patient interviews. Assessed for face and content validity by 2 clinical pharmacists and 2 healthcare researchers.  Minor revisions in wording and structuring of some items after small patient pilot	No data presented	
The patterns of asthma medication use questionnaire [87]	5	Dichotomous and likert items	None specified	Yes	Developed with reference to research literature, qualitative work and discussions of an expert panel	No data presented	
Visual Analogue Scale (VAS) 1 week version [53]	1	Continuous (visual analogue)	Previous week	No	No description of development	No data presented	
Visual Analogue Scale (VAS) one month version [88]	1	Continuous (visual analogue)	Previous month 	No	No description of development	No data presented	
Visual Analogue Scale (VAS) six month version [89]	1	Continuous (visual analogue)	Previous 6 months	No	No description of development	No data presented	
Visual Analogue Scale (VAS) carers' version [90]	1	Continuous (visual analogue)		No	No description of development	No data presented	
Table 1 Properties of adherence scales
NB version names have been used here for distinction and were not provided by authors of measures.
