# Supplementary material for: Suitability of measures of self-reported medication adherence for routine clinical use: A systematic review
Source: BMC Med Res Methodol. 2011 Nov 3;11:149. doi: 10.1186/1471-2288-11-149 (PMC3219622; doi:10.1186/1471-2288-11-149)
Supplement: Additional file 2 — Validity and reliability of self reported adherence scales. A table giving details of validity and reliability studies on included measures. [file 1471-2288-11-149-S2.RTF]

	Validity	Reliability	
Scale	Ref	Sample size	Population	Sampling strategy	Validated against	Validity Results		
AACTG
4 day version	15	93	Individuals over 12 with documented HIV-1 infection 	Not stated	HIV1-RNA 	Significant association (p=0.03)	Not reported	
					CD4 count	No significant association (no p value reported)	Not reported	
	16	119	HIV positive adults	Convenience sample	MEMS	Significant difference (p=<0.01)	Not reported	
	17	211	HIV infected adults	Not stated	Pill count	No significant correlation(p>0.06)	Not reported	
		61			MEMS	No significant correlation (p0.05-0.06)	Not reported	
		224			Change in HIV RNA	Significant correlation 
Pearson = 0.150-0.177 (p<0.05)	Not reported	
	18	323	HIV positive adults	Not stated	MEMS	Correlation range from -0.07 to -0.25.  No p values reported	Not reported	
					Viral load	Correlation range from 0 to -0.28.  No p values reported	Not reported


	
AACTG 3 day version
	19	84	Patients with HIV	Not stated	Unannounced pill count	No significant correlation r=0.71 (p=0.52)	Not reported	
					Viral load	No significant correlation 
r= -0.34 (p=0.22)	Not reported	
	20	524	Patients over 18 with HIV	Not  stated	Viral load	Only significantly correlated at ¼ time points (p<0.05)	Not reported	
	21	34	Antiretroviral-naive patients	Not stated	MEMS	Significant correlation Spearman= 0.87 (p=<0.0001)	Not reported	
					Pill count	Significant correlation Spearman= 0.89 (p=<0.0001)	Not reported	
					Viral load	Significant correlation Spearman= -0.42(p=0.01)


	Not reported	
AACTG last 2 day + Saturday version	22	46	Mothers with HIV with well children	Not stated	Pill Count	Significant association Kappa = 0.401 (p=<0.001)	Not reported	
					Viral load	 Significant association (p<0.05)	Not reported	
					CD4	Significant association (p<0.05)	Not reported	
AACTG 7 day version	23	474	Patients with HIV	Not stated	HIV RNA	Significant association (p<0.01)	Not reported	
AACTG one month version	24	114	HIV infected adolescents aged 12-19 who were infected through sex or drug taking behaviour
	Not stated	HIV viral load	 Significant association (p=0.02)	Not reported	
AACTG Reasons subscale
	90	240	Patients over 18 with HIV 	Convenience sample	Not reported	Not reported	Cronbach's alpha = 0.89


	
Adherence Self Report Questionnaire (ASRQ)	25	78	Outpatients over 18 with hypertension, diabetes or dyslipidemia 	Not stated	MEMS	No significant association (no p value reported) 	Not reported	
	26	245	Patients from GP practice taking antihypertensives	Not  stated	MEMS	 Significant association (p=0.0004)	Not reported	
	27	216	Patients from GP Practices taking antihypertensives	Not stated	MEMS	Sensitivity=46%
Specificity=66%	Not reported	
Adherence to Refills and Medication Scale (ARMS)	28	435	Patients with coronary heart disease	Not stated	Refill adherence	Significant correlation r=0.323 (p=<0.01)	Cronbach's alpha= 0.814
	
		429	Patients with hypertension	Not stated	Diastolic blood pressure	 Significant association (p=<0.05)	Not reported	
					Systolic blood pressure	No significant association (no p value reported)	Not reported


	
Ask 12 behaviour subscale	29	93	Patients over 18 with a diagnosis of asthma, diabetes or congestive heart failure	Not stated	Pharmacy refill	No significant correlation (p>0.06)		
		106			SF-12 physical component (generic health status questionnaire)	No significant correlation (p>0.06) 		
		67			ADS Score (scale assessing impact of diabetes on patients' life)	Significant correlation Spearman correlation= 0.31 (p= 0.05)		
		41			Mini AQLQ score (measure of quality of life in asthma)	No significant correlation (p>0.06)		
		112					Cronbach's's alpha=0.61
Test-retest reliability ICC= 0.67


	
Barroso	30	93	HIV infected men	Not stated	Seminal HIV suppression	Significant association (p=0.013)	Not reported	
					HIV viral load	Significant association (p=0.001) 	Not reported	
Bell	31	80	HIV positive patients over 16	Not stated	MEMS	No significant association (p=0.67)	Not reported	
Brooks Medication Adherence 
Scale	32	263	Adults with asthma	Not stated	Not reported	Not reported	Cronbach's's alpha = 0.76	
		232					Cronbach's's alpha = 0.8	
Choo	33	286	Patients over 18 who had hypertension	Not stated	Refill rate	For items 1,2,3,and5 (P=0.001-0.02)	Not reported	
	34	75	Patients over 18 who had received warfarin for over 6 months	Not stated	Refill rate	R=0.465 (p=<0.001)	Not reported	
					INR within therapeutic range	R=0.314 (p=0.006)	Not reported	
Cohn	35	643	Patients with HIV	Not Stated	Adverse events and death	Significant association (p<0.01)	Not reported	
Composite self report measure	36	319	Home Care clients over 65	Random sample	Pill count	66.3% agreement Kappa = 0.29	Not reported


	
CPCRA antiretroviral medication self report- 3day	37	64	Patients with drug resistant HIV-1	Random Sample	HIV RNA	No significant association (p value not reported)	Not reported	
					CD4 count	No significant association(p value not reported)	Not reported	
CPCRA antiretroviral medication self report- 7 day	38	540	HIV infected persons	Not stated	HIV RNA	 Significant association (p<0.001)	Not reported	
	37	70	Patients with drug resistant HIV-1	Random Sample	HIV RNA	No significant association(p value not reported)	Not reported	
					CD4 count	No significant association(p value not reported)	Not reported	
Erickson	39	100	Patients with asthma	Not stated	Pharmacy refill data	Significant correlation
Pearson = 0.382 (p,0.001)
	Cronbach's's alpha = 0.86	
Fodor	40	359	Blue collar workers with hypertension	Not stated	Systolic blood pressure	Significant association (p=<0.002)	Not reported	
					Diastolic blood pressure	Significant association (p=<0.01)	Not reported	
Gehi	41	1015	Outpatients with documented chronic heart disease	Not stated	Development of cardiovascular events	Significant association (p=0.03) bivariate analysis, 0.06 multivariate analysis)	Not reported	
Grymonpre	42	135	Patients over 65	Not stated	Pharmacy claims data	No significant difference (p value not reported)	Not reported	
Hill Briggs 	43	181	African Americans with type 2 diabetes	Not stated	AIC levels (a measure of diabetes control)	 Significant correlation (p=0.0001).
Items 1 and 2 (running out of medication and forgetting to take medication significantly correlated (p=<0.01, <0.05 respectively).  Items 3-5 (carelessness, stopping because better or worse) not significantly correlated (p>0.05). 

	Not reported	
Immunosupressant 
Therapy Adherence Scale (ITAS) 	44	66	Transplant recipients	Not stated	IS blood levels	Significant correlation= 0.52 (p=0.01)	Not reported	
					Refill rate	Significant correlation= 0.57 (p=0.01)	Not reported	
	45	102	Transplant recipients	Not stated			Cronbach's's alpha=0.81	
Inui	46	241	Patients with hypertension	Not stated	Pill count	Sensitivity = 55%
Specificity = 88%	Not reported	
Kerr	47	88	Injection drug users	Not stated	Pharmacy refill records	No significant association (p=0.5)	Not reported	
Leopold	48	40	Patients taking antiparkinson medication at least 3 times daily 	Not stated	MEMS	 Significant difference(p=<0.01)	Not reported	
Liu	49	330	Patients taking protease inhibitors or non nucleoside reverse transcriptor 	Not stated	MEMS	90 % adherence:
Sensitivity 23.89%
Specificity 93.91%
95% adherence:
Sensitivity = 30.9%
Specificity = 93.02%


	Not reported	
Lu 1month frequency scale	50	156	Patients with HIV	Not stated	MEMS	 Significantly different(p<0.01)	Not reported	
					HIV RNA	Significant association (p=<0.05)	Not reported	
Lu 1 month percent scale	50	156	Patients with HIV	Not stated	MEMS	 Significantly different(p<0.01)	Not reported	
					HIV RNA	 Significant association (p=<0.05)	Not reported	
Lu 1 month rating response scale	50	156	Patients with HIV	Not stated	MEMS	Not significantly different (p>0.05)	Not reported	
					HIV RNA	 Significant association (p=<0.05)	Not reported	
Multicentre aids cohort study (MACS) adherence form  	51	393	HIV positive men	Not stated	HIV RNA	 Significant association (p= 0.013)


	Not reported	
MARS	52	58	Renal transplant patients over 18	Stratified random sample	MEMS	Sensitivity= 57.1%
Specificity= 70.6%	Not reported	
	53	280	Patients with schizophrenia and bipolar disorder	Not stated	Psychotropic concentration dose ratio	Significant association Spearman = 0.52 (p=0.01)
Sensitivity 98%
Specificity 33%	Cronbach's's alpha=0.78	
Medication Adherence Evaluation Scale General Inquiry (MASS)	54	22	Patients with psychiatric disorder	Not stated	Not reported	Not reported	Cronbach's alpha= 0.9	
MATI


	18	323	HIV positive adults	Not stated	MEMS	Correlation range from 0.19 to 0.40.  No p values reported	Not reported	
					Viral load	Correlation range from -0.15 to -0.32.  No p values reported


	Not reported	
Medication adherence self report inventory (MASRI) part A	55	55	Patients over 16 with SLE	Convenience Sample	Pill counts	No significant correlation (p=0.53-0.58)	Cronbach's alpha = 0.7	
					Pharmacy	Significant correlation)Spearman correlation =0.55-0.62 (p<0.001-0.002)		
		20					Test-retest reliability: 
Kappa  for likert scale items =0.5
 ICC for continuous (visual analogue) item = 0.93	
	56	78	Patients with HIV-1	Not stated	MEMS	Significant Pearson correlation 3 days adherence = 0.32 (p=0.004), 2 weeks = 0.62 (p=<0.001) 1 month (visual analogue) 0.63(=<0.001)	Not reported	
					Viral load	Significant correlation sigma= -0.37(p=0.001)

	Not reported	
Medical Outcomes Study  (MOS)	57	139	Patients over 18 with hypertension, diabetes mellitus, hypercholesterolemia, hypothyroidism or condition requiring HRT	Not stated	Pharmacy refill records	Spearman Rho= 0.261(p=0.05)	Not reported	
Melbourne	58	44	English speaking HIV patients	Not stated	MEMS	97% self report vs 90.3% MEMS (no comparative statistics)	Not reported	
Mooney	59	55	Patients between 18 and 55 with cocaine dependence	Not stated	MEMS	 Significant difference(p=0.0001)	Not reported	
					Riboflavin level	 Significant difference (p=0.0267)	Not reported	
Morisky 4	60	290	Outpatients with high blood pressure	Random Sample	Blood pressure control	Significant correlation r=0.58 (p<0.01)
Sensitivity = 81%
Specificity = 44%	Cronbach's alpha = 0.61	
	61	260	HIV infected persons over 18 attending hospital outpatient clinics	Not stated	Not reported	Not reported	Cronbach's alpha = 0.31	
	62	420	Patients over 18 infected with HIV 	Convenience sample	Not reported	Not reported	Cronbach's alpha = 0.71


	
	63	153	Patients over 18 with a confirmed diagnosis of Crohns disease or ulcerative colitis	Convenience sample	Not reported	Not reported	Cronbach's alpha= 0.5
Intentional non adherence Cronbach's alpha = 0.52.
Unintentional non adherence Cronbach's alpha =0.54	
	64	93	Outpatients with a psychiatric disorder other than schizophrenia, dementia, alcoholism and mental retardation	Not stated	Not reported	Not reported	Cronbach's alpha =0.59-0.71	
	65	377	Patients who had taken an ace inhibitor or lipid lowering agent for at least 3 consecutive months	Not stated	Prescription refill data	Significant correlation r=0.3( p=0.001)	Cronbach's alpha = 0.32	
	66	82	Patients 18 or over commencing treatment on dothiepin or amitryptiline	Convenience sample	MEMS	Sensitivity =72.2%
Specificity = 74.1%
For >80% compliance	Not reported	
	27	139	Patients over 18 with hypertension, diabetes mellitus, hypercholesterolemia, hypothyroidism or condition requiring HRT	Not stated	Pharmacy refill records	Significant correlation Spearman Rho= 0.234 (p=0.05)	Not reported	
	36	319	Home Care clients over 65	Random sample	Pill count	62.5% agreement Kappa =0.13	Cronbach's alpha = 0.42	
	52	58	Renal transplant patients over 18	Stratified random sample	MEMS	Sensitivity= 57.1%
Specificity= 68%
For >80% compliance	Not reported	
	67	109	Patients with high blood pressure	Random Sample	Pill Count	No significant association (p=0.36):
Sensitivity 73.5%
Specificity 45.3%	Not reported	
	68	30	Patients over 15 with pulmonary tuberculosis	Not stated	MEMS	Sensitivity =  69%
Specificity = 76%	Not reported	
	90	180-240	Patients with HIV	Convenience Sample	Pill count	Significant correlation phi= 0.14-0.29 (p<0.05)	Cronbach's alpha =0.79	
					Pharmacy refill	Significant correlation phi=0.16 p<0.05)		
Morisky 4 likert	36	319	Home Care clients over 65	Random sample	Pill count	58.4% agreement Kappa = 0.08 	Cronbach's alpha =0.42	
Morisky 8	69	1367	Patients with hypertension	Random sample	Blood pressure control	Significant association (p<0.05)
Sensitivity = 93%
Specificity = 53%	PCA- factors loaded onto a single factor	
	70	87	Older adults with hypertension	Stratified random sample	Pharmacy refill data	 Significant association (p=0.001)	Not reported	
Murphy 1	71	161	HIV positive adolescents aged between 13-18.	Not stated	HIV viral load	Significant association (p,0.001)	Not reported	
Murphy 2	72	46	Mothers with HIV with well children	Not stated	Pill Count	Significant association Kappa = 0.522 (p=<0.001)	Not reported	
					Viral load	No significant association (p>0.1)	Not reported	
					CD4	No significant association (p>0.1)	Not reported	
Prado	67	109	Patients with high blood pressure	Random Sample	Pill Count	No significant association:
Sensitivity 88.2%
Specificity 18.7%	Not reported	
Pratt	73	69	Patients over 50 with serious mental illness other than dementia	Not stated	Pill Count	No significant correlation (p>0.05)
	Not reported	
Reported adherence to medication scale (RAM)	61	260	HIV infected persons over 18 attending hospital outpatient clinics.  	Not stated	Not reported	Not reported	Cronbach's alpha = 0.66	
	74	524	Patients with asthma or diabetes, psychiatric patients, cardiac, general medical and renal in-patients.	Not stated	Not reported	Not reported	Cronbach's alpha = 0.6-0.83	
Regimen screen of Brief Medication Questionnaire (BMQ)	57	139	Patients over 18 with hypertension, diabetes mellitus, hypercholesterolemia, hypothyroidism or condition requiring HRT	Not stated	Pharmacy refill records	No significant correlation (p>0.05)	Not reported	
	75	22			MEMS	Sensitivity = 80%
Specificity =100%f or>80% adherence	Not reported	
Remington	76	52	Patients with schizophrenia aged between 18-65 	Not stated	MEMS	No significant correlation (However, approaching significance (p=0.06)	Not reported	
Schneider	77	392	Patients with HIV	Purposive sample	Viral load	Significant association (p= 0.03)		
		552					Cronbach's alpha = 0.75	
Schuman	78	453	HIV seropositive women	Not stated	CD4 count	 Significant association (p=0.038)	Not reported	
					HIV viral load	Significant association (p=0.001)

	Not reported	
Self Reported Adherence (SERAD)	79	530	HIV outpatients over 18 taking HAART	Not stated	Pill count	No significant difference (no p value reported)	Not reported	
					MEMS	No significant difference (no p value reported)	Not reported	
Simplified Medication Adherence Questionnaire (SMAQ)	80	40
	Patients with HIV taking nelfinavir in combination with other antiretroviral drugs	Not stated	MEMS	Sensitivity 72%
Specificity 91%		
		1797			Viral load	 Significant association (p=0.0001)		
		1376					Cronbach's alpha=0.75
Inter observer reliability kappa= 0.74	
	81	85	HIV infected persons over 18	Not stated	Viral load	Significant association (p0.005)


	Not reported	
SR Recall	82	298	Persons over 18  living with HIV	Convenience sample	Unannounced pill count	Significant difference (p=<0.01). Pearson correlation =0.34
.  K coefficient range = 0.09-0.25
 	Not reported	
					Viral load	 Significant correlation (p=<0.05)	Not reported	
Stages of Change (SOC)	83	85	HIV positive patients taking protease inhibitors aged between 18-74	Not stated	MEMS	 Significant association (p=0.03)	Not reported	
	84	22	HIV infected low income patients	Not stated	MEMS	Significant association 16 weeks after starting medication (p<0.001)	Not reported	
Stewart	85	98	Primary care patients other than those aged between 8 and 18	Convenience sample	Pill count	Sensitivity= 80%
Specificity= 69.8%	Not reported	
Tool for Adherence Behaviour Screening (TABS)	86	276	Patients with chronic lung conditions	Not stated	Not reported	Not reported	Cronbach's alpha 'adherence' = 0.80
Cronbach's alpha 'non adherence' = 0.59	
		20					Test retest reliability:
ICC 'adherence' = 0.78
ICC 'non adherence.' = 0.91	
The patterns of asthma medication use questionnaire	87	176	Patients with asthma aged 20-60	Not stated	Asthma severity	No significant association (p=0.24)	Not reported	
					Unscheduled healthcare visits	 Significant association (p<0.005)	Not reported	
Visual Analogue Scale (VAS) 1 week version 	53	280	Patients with schizophrenia and bipolar disorder	Not stated	Unannounced pill count	Significant correlation Pearson correlation = 0.48.  (p=<0.01K coefficient range = 0.32-0.49	Not reported	
Visual Analogue Scale (VAS) 1 month version	82	298	Persons over 18  living with HIV	Convenience sample	Viral load	Significant correlation (p=<0.01)	Not reported	
					Pharmacy records	Spearman correlation= 0.19 	Not reported	
	88	178	Patients over 16 with HIV who had been prescribed HAART for the past month 	Not stated	Unannounced pill count	No significant correlation r=0.76 (p=0.52)	Not reported	
	19	84	Patients with HIV	Not stated	Viral load	No significant correlation (p=0.14-0.96)	Not reported	
					MEMS	Significant correlation Spearman =  0.29(p=0.018)	Not reported	
	25	78	Outpatients over 18 with hypertension, diabetes or dyslipidemia 	Not stated	MEMS	Significant correlation Spearman= 0.77 (p=<0.0001)	Not reported	
	21	34	Antiretroviral-naive patients	Not stated	Pill count	Significant correlation Spearman= 0.86 (p=<0.0001)	Not reported	
					Viral load	Significant correlation Spearman= -0.36(p=0.03)


	Not reported	
					MEMS	Pearson correlation 3 days adherence = 0.32 (p=0.004), 2 weeks = 0.62 (p=<0.001) 1 month (visual analogue) 0.63(=<0.001)	Not reported	
	56	78	Patients with HIV-1	Not stated	Viral load	Significant correlation sigma= -0.37(p=0.001)	Not reported	
					Pharmacy refill data	Significant correlation Pearson =0.22-0.26 (p=<0.01)	Not reported	
Visual Analogue Scale (VAS) 6 month version	89	1985	Patients over 18 with diabetes	Not stated	MEMS	VAS higher than MEMS adherence mean difference 15% (p value not reported)	Not reported	
Visual Analogue Scale (VAS) carers' version	90	73	Carers of patients with HIV	Not stated	Viral load	For 90% adherence:
Sensitivity = 100%
Specificity = 26.3%	Not reported	
Table 2:  Validity and Reliability of self reported adherence scales
NB version names have been used here for distinction and were not provided by authors of measures.
